# The Role of Ambient Ozone in Epidemiologic Studies of Heat-Related Mortality

**DOI:** 10.1289/ehp.1205251

**Published:** 2012-08-16

**Authors:** Colleen E. Reid, Jonathan M. Snowden, Caitlin Kontgis, Ira B. Tager

**Affiliations:** 1 Department of Environmental Health Sciences, and; 2 Division of Epidemiology, School of Public Health, University of California, Berkeley, Berkeley, California, USA; 3 Department of Obstetrics and Gynecology, Oregon Health and Science University, Portland, Oregon, USA; 4 Department of Geography, Center for Sustainability and the Global Environment, University of Wisconsin–Madison, Madison, Wisconsin, USA

**Keywords:** causality, confounding variables, epidemiology, extreme heat, mortality, ozone

## Abstract

Background: A large and growing literature investigating the role of extreme heat on mortality has conceptualized the role of ambient ozone in various ways, sometimes treating it as a confounder, sometimes as an effect modifier, and sometimes as a co-exposure. Thus, there is a lack of consensus about the roles that temperature and ozone together play in causing mortality.

Objectives: We applied directed acyclic graphs (DAGs) to the topic of heat-related mortality to graphically represent the subject matter behind the research questions and to provide insight on the analytical options available.

Discussion: On the basis of the subject matter encoded in the graphs, we assert that the role of ozone in studies of temperature and mortality is a causal intermediate that is affected by temperature and that can also affect mortality, rather than a confounder.

Conclusions: We discuss possible questions of interest implied by this causal structure and propose areas of future work to further clarify the role of air pollutants in epidemiologic studies of extreme temperature.

There is a large and growing literature investigating the role of extreme heat on mortality. Many of these studies have found a significant increase in mortality associated with hot days (Basu 2009). Tropospheric ozone, which is positively and often highly correlated with temperature in most locations throughout the world, is a secondary pollutant generated through photochemical reactions involving precursors such as oxides of nitrogen (NO_x_), carbon monoxide, and volatile organic compounds (VOCs) (Stockwell et al. 1997). Ozone exposure has been associated with an array of health outcomes, including pulmonary function, hospital admissions, and daily mortality. Researchers have conceptualized the role of ozone in studies of heat in various ways—as a confounder (e.g., Basu et al. 2005; Hajat et al. 2002), as an effect modifier (e.g., Rainham et al. 2005; Ren et al. 2008), and as a co-exposure (e.g., Katsouyanni et al. 1993; Sartor et al. 1995)—reflecting a lack of consensus as to how to account for ozone effects.

Directed acyclic graphs (DAGs) are tools that are employed with increasing frequency in epidemiology for encoding subject matter knowledge, guiding data collection, verifying identifiability, and informing analysis (Greenland et al. 1999). DAGs enable researchers to visually and explicitly represent current knowledge about a given topic and the hypothesized exposure–outcome relation to be investigated. Causal graphs encode the specific mechanistic system that can be expressed as nonparametric structural equations that are at the core of the research question and present a graphical analog to epidemiologic concepts such as confounding, selection bias, and direct/indirect causal effects. DAGs can complement many analyses by encouraging the researcher to explicitly state the hypothesized associations and causal pathways among exposure, confounders, outcome, and other covariates (Hernan et al. 2002), and thus DAGs provide guidance for analytical decisions.

Here, we apply DAGs to the topic of heat-related mortality to provide insight into the causal structure of the heat–ozone–mortality relationship. After summarizing the existing literature on heat and ozone, we represent the variables using causal graphs and apply this information to discuss the analytical implications of each approach. The primary outcome discussed is mortality—the most common outcome analyzed in previous studies of extreme heat exposure. Whereas prior research on the health effects of heat has considered additional ambient air pollutants, here we focus on ozone because of its consistent association with temperature across locations. After applying DAGs and knowledge of the subject matter, we propose a causal structure for this topic and discuss how the appropriate method of analysis depends on the specific scientific question that one wishes to address, and the extent to which assumptions are met.

## Role of Ozone in Studies of Temperature-Related Mortality

Among the studies that have dealt with ozone as a third variable in the heat–mortality association, the motivations and explanations vary. Many papers examining the effect of heat on mortality controlled for ozone as a confounder without further discussion of the motivation for such designation or investigation of the quantitative effect of including ozone in their statistical models (e.g., Hajat et al. 2007; Ishigami et al. 2008). Some studies have stated that ozone confounds the relationship between temperature and mortality, because ozone formation is dependent on temperature (e.g., Anderson and Bell 2009), because air pollution is a function of atmospheric conditions (e.g., Rainham and Smoyer-Tomic 2003), or because ozone and temperature have been found to be statistically correlated (e.g., Basu 2009). Other studies did not state explicitly why they included ozone in their statistical models. Of these, the authors either stated that they controlled for ozone as a confounder without further elaboration (e.g., Goldberg et al. 2011) or they added ozone to their models without stating the causal relationships among temperature, ozone, and mortality (e.g., Kovats et al. 2004).

Recently, many studies have treated ozone not only as a confounder of the temperature–mortality relationship, but also have investigated to what extent ozone confounds this relation. Most of these studies have assessed the strength and nature of confounding by fitting models with and without ozone. For some studies, this “add in/take out” approach revealed that including ozone did not meaningfully alter the effect estimates for the daily heat–mortality relationships (e.g., Basu et al. 2005; Hajat et al. 2002; Stafoggia et al. 2006). In other studies, adding ozone to the models slightly decreased the effect estimates for temperature and mortality (e.g., Bell et al. 2008; O’Neill et al. 2005; Rainham and Smoyer-Tomic 2003). If ozone is in fact on the causal pathway between temperature and mortality (which we posit below), controlling for ozone in a statistical model estimates the controlled direct effect of temperature on mortality, under certain assumptions (Petersen et al. 2006). The controlled direct effect is the effect of temperature through pathways that do not pass through ozone, holding ozone at a constant level across all individuals.

Interestingly, some of the earliest papers on the topic of heat and mortality treated ozone as a co-exposure with temperature rather than as a confounder. Katsouyanni et al. (1993) and Sartor et al. (1995) found significant interaction between ozone and temperature. In the discussion of their findings, Sartor et al. (1995) noted that high temperatures do affect ozone formation, such that part of the observed effect could be attributable to indirect rather than direct effects of temperature. Subsequently, they dismissed this hypothesis because of reasoning that does not conform to current understanding of the subject matter.

More recent studies have also assessed effect modification between ozone and temperature on mortality. An analysis of the U.S. National Morbidity, Mortality, and Air Pollution Study data found that ozone significantly modified the association between maximum temperature and cardiovascular mortality in 95 U.S. communities, and, when stratified by region of the country, strong interactions occurred in most regions (Ren et al. 2008). However, this result could be attributable to regional differences in population susceptibility or air pollution mixtures. Another analysis found no significant interactions between ozone and synoptic air masses, which are hypothesized to correspond more closely to physiological responses to heat than do individual measures of weather such as temperature (Rainham et al. 2005). And only 2 of 15 British cities demonstrated significant effect modification between ozone and temperature during a decade of summers (Pattenden et al. 2010).

Additionally, some studies have investigated both confounding and effect modification by ozone. Basu et al. (2008) found borderline significant effect modification by ozone in two counties, but none in their combined analysis of nine counties in California, and they found no evidence of confounding by ozone. Zanobetti and Schwartz (2008) found no effect modification by ozone in nine U.S. cities, but did find that including ozone as a confounder slightly decreased the effect estimate for heat on mortality, similar to other studies mentioned above.

Still another approach for dealing with ozone in studies of effects of temperature on mortality is to not control for it. Although many studies do not control for ozone for unspecified reasons (e.g., Barnett 2007), in recent years some authors have argued that ozone or other pollutants may be on the causal pathway between temperature and mortality, and have questioned the appropriateness of controlling for it as a confounder on this basis (Goldberg et al. 2011; McMichael et al. 2008). Others have not controlled for ozone because previous analyses empirically demonstrated that ozone was not a confounder, rather than because of posited causal relationships among the variables (Anderson and Bell 2011). The assertion that ozone does not confound the heat–mortality relationship was also stated by Martiello and Giacchi (2010) based on their recent review of the health effects of heat on both morbidity and mortality. Assuming that ozone is on the causal pathway between temperature and mortality, studies that do not control for ozone estimate the total effect of temperature on mortality, through both direct and indirect pathways with respect to ozone.

Overall, there is a lack of consensus about the roles that temperature and ozone play, interrelatedly, in causing mortality. This inconsistency may be explained by different understandings of the subject matter or different formulations of the question of interest. Yet the determination of whether ozone is a confounder, an effect modifier, or a causal intermediate of the heat–mortality association is critical to valid analysis and meaningful interpretation of findings. To elucidate these methodological issues and ground them in the subject matter, we employ DAGs to graphically represent the causal structures of temperature, ozone, and mortality. In doing so, we aim to inform future studies of temperature and mortality and also to enable comparison of various approaches existing in the literature.

## Causal DAGs of Heat, Mortality, and Ozone

**Figure 1:**
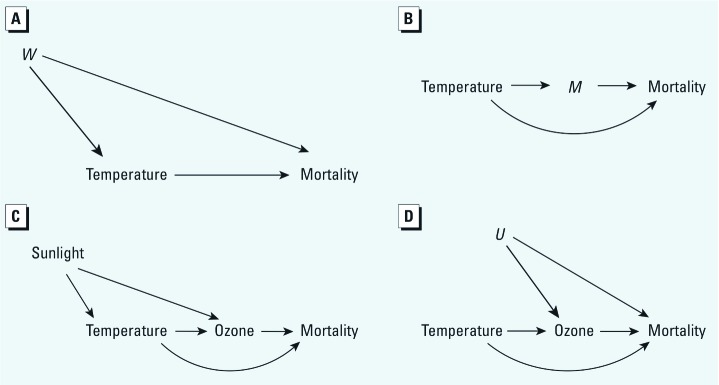
DAGs demonstrating the research question of the relations among temperature, ozone, and mortality. (*A*) A generic representation of confounding; *W* is a confounder of the temperature–mortality association because it predicts both variables. (*B*) A demonstration of a causal effect mediator *M*; ­temperature may have both a direct effect unmediated by *M* and an indirect effect, of which *M* is on the causal pathway. (*C*) A DAG of the posited role of ozone in the temperature–mortality association, with sunlight a predictor of both ambient temperature and tropospheric ozone formation. This DAG simplifies to the same structure as (*B*). (*D*) If the ozone–mortality association is confounded (here by factor *U*), then ozone is a collider, and controlling for it biases the temperature–mortality association.

Figure 1A and B presents two generalized possible causal structures for the association between extreme heat and mortality; this effect of interest is shown by the directed arrows from temperature to mortality. Figure 1A shows the role of a hypothetical confounder, *W,* in this relationship. In accordance with the definition of a confounder, *W* is associated with both exposure (temperature) and outcome (mortality) and provides an unblocked backdoor path between mortality and temperature, in the language of DAGs (Greenland et al. 1999). A variable need not actually “cause” the exposure in order to introduce confounding bias (Rothman et al. 2008). One firm requirement of a confounder, however, is that it must not be on the causal pathway between exposure and outcome, as *M* is in Figure 1B. This DAG represents two causal pathways between temperature and mortality, one indirect effect mediated by *M*, and one direct effect (which is direct with respect to *M*—i.e., unmediated by this particular variable). In such a scenario, *M* is termed a causal intermediate or a mediator of the effect of temperature on mortality. As has been repeatedly noted, such a variable is not extraneous to the effect of interest in the way that a confounder is, but is instead a component of that effect and requires analytical techniques that may be distinct from those used for confounder control (Rothman et al. 2008). Although the concepts of confounding and mediation have long been defined in health sciences, in practice the two structures have often been conflated [for discussion of one example, see Schisterman et al. (2009)]. This owes partly to the application of purely statistical definitions of confounding (e.g., the criterion of “substantial magnitude change” of the effect estimate when controlling for a third variable), under which both causal mediation and selection bias will often meet the definition of a confounder (Hernan et al. 2002), blurring the distinctions among these distinct causal structures.

Analysis of the present research question should be dictated by hypotheses on how ozone is causally related to temperature and mortality: in the place of *W,* a confounder, or, in the place of *M,* a causal intermediate. If ozone is a confounder of the temperature–mortality relationship, it needs to cause both temperature and mortality. Although ozone can cause mortality (Jerrett et al. 2009; Thurston and Ito 2001), we are aware of no evidence that ozone levels have an impact on local temperature sufficient to provoke a health response. Therefore, Figure 1A is not likely plausible. Temperature and ozone are highly correlated, partly because the formation of ozone is dependent on sunlight, which leads to increases in ambient temperatures, and partly because temperature determines transformations of primary emissions, formation of precursors to ozone formation, and their transport processes (Stockwell et al. 1997). Based on the atmospheric chemistry of ozone, Figure 1B is the more appropriate causal model. Temperature and the factors associated with it predict ozone concentrations, which, in turn, predict mortality (an indirect effect of temperature on mortality), and temperature increases mortality aside from the ozone-mediated pathway (i.e., a direct effect). Under this framework, ambient ozone is a causal intermediate of the association between temperature and mortality—*M* in Figure 1B. The DAG that we propose for the relationship of temperature on mortality is shown in Figure 1C, which reduces to Figure 1B.

## Analysis Informed by the Causal Question

At least three epidemiologic questions of interest are suggested by our DAG. We conceptually discuss these questions and refer readers elsewhere for details on estimation. First, we may be interested in the total effect of temperature on mortality through all causal pathways (i.e., the combined effect of the direct and indirect pathways). Second, we may want to estimate the direct effect of temperature on mortality (i.e., the effect not mediated through the intermediate, ozone). Third, we may be interested only in the indirect effects of temperature through the intermediate, ozone, to understand the fraction of the temperature effect attributable to ozone.

To address the first question, that of the total effects of temperature on mortality, an investigator should not adjust for ozone for valid health effects estimation. The strong correlation between temperature and ambient ozone that is empirically observed in many regions does not pose a threat to the validity of total health effects calculations; however, when decomposing effects into direct and indirect effects, positivity problems may arise. The total effect of temperature estimates the complete effect of extreme heat on health, which includes the fact that ozone levels are affected by temperature. These estimates may be useful for risk assessments of health effects of future heat events under various climate change scenarios (though future changes in air pollution, climatic conditions, and demographic and social factors may alter the total effects of heat in unpredictable ways).

For the second question, that of the direct effect of temperature on mortality not mediated through ozone, multiple approaches for estimation exist (Petersen et al. 2006; VanderWeele 2009). Two kinds of direct effects exist. The controlled direct effect describes the non-ozone-mediated effects of temperature on mortality when ozone is held constant across all independent study units (e.g., individuals or cities). The natural direct effect of temperature on mortality describes the non-ozone-mediated effects when ozone is set to the level that would have occurred if there were no effect of temperature on ozone, such as some background ozone level. In the absence of interaction between the exposure and the intermediate, the controlled and natural direct effects are equivalent. However, if effects of temperature vary across strata of ozone, then the estimation of natural direct effects is often more complex than the techniques for controlled direct effects (Petersen et al. 2006). Both controlled direct effects and natural direct effects have policy implications because they estimate the part of the temperature effect that impacts health separate from the effects through ozone (curved line in Figure 1C). The controlled direct effect is useful for estimating the effect of temperature on health under various proposed uniform ozone standards because it estimates the effect of temperature on mortality holding ozone constant at the same value for all study units. U.S. ozone standards are nationally uniform; however, many locations are currently out of compliance, and more locations may be out of compliance in the future due to increasing temperatures from climate change affecting ozone levels. To estimate the effect of temperature on mortality under realistic future scenarios of temperature, the natural direct effects may give a more realistic estimate because they take into account variations in ozone among study units due to geography and natural differences, while holding ozone constant within a study unit.

Controlled direct effects can be calculated simply by including the intermediate in a standard regression, a long-applied approach in epidemiology (Rothman et al. 2008), as long as confounders of both the temperature–mortality relationship (*W* in Figure 1A) and the ozone-mortality relationship (*U* in Figure 1D) are accounted for in the model (VanderWeele 2009). In the presence of a factor that causes ozone and mortality (e.g., NO_x_), ozone is a collider because both *U* and temperature cause ozone. Controlling for a collider to block the indirect pathway to obtain the controlled direct effect in this scenario has the unintended consequence of distorting the effect of interest between temperature and mortality (Cole and Hernan 2002; Schisterman et al. 2009).

The third question—that of the indirect effect of temperature on mortality mediated through ozone—can be estimated by subtracting the natural direct effect from the total effect, yielding the natural indirect effect (VanderWeele 2009), or by estimating the two steps of the effect of changes in temperature on ozone and then the effects of those changes in ozone on mortality (Pearl 2011). The indirect effect estimates the proportion of the total effect of temperature that is attributable to ozone. In many heat wave events, both ozone and temperature levels are high, and proper attribution of the deaths to each exposure has been attempted (e.g., Filleul et al. 2006). Better estimation of the deaths attributable to ozone versus temperature can be helpful for setting policy and planning interventions to prevent deaths during future heat waves.

A final consideration when estimating temperature effects within or across strata of ozone is the assumption of positivity. This assumption requires that there be a positive probability of each exposure level whose effects are calculated across all joint strata of the covariates. The positivity assumption requires particular attention in the presence of continuous and/or correlated exposures and covariates (Petersen et al. 2010). For a heuristic example using binary exposures (temperature: high/low; ozone: high/low), if a data set contains no high ozone levels on days of low temperature, then the effects of temperature are not identifiable within all strata of ozone, and the resulting calculation will be biased. Although the actual decomposition of temperature and ozone effects is more complex, this example demonstrates the stronger assumptions taken on when estimating effects of complex, multivariable exposures (compared with the total effects, which require positivity across strata of confounders but not across strata of the mediator, ozone).

## Additional Causal Structure and Variables

Figure 1C, does not represent the full complexity that characterizes the relations among temperature, ozone, and mortality. Precursors of ozone including NO_x_ and VOCs, and the co-exposure of particulate matter, another component of smog, could be introduced to the DAG. These additional considerations might lead to a very complex DAG that is beyond the scope of this commentary, but could be of interest for future research. Additionally, although effect modification cannot be represented using DAGs, causal intermediates may still be effect modifiers, and the calculation of natural direct/indirect effects also enables assessment of effect modification (Petersen et al. 2006).

## Conclusion

On the basis of the subject matter encoded in our DAGs, we assert that the role of ozone in the causal structure of temperature and mortality is a causal intermediate rather than a confounder. Ozone is affected by temperature and can also affect mortality, placing it on the causal pathway between exposure and outcome. This causal structure lends itself to multiple questions of interest, including the total effects of temperature, the direct effects of temperature not mediated through ozone, and the indirect effects of temperature through ozone. Each question has a respective application, and the choice of total versus direct/indirect effects requires different analytical techniques. We have summarized the theoretical approaches and assumptions underlying these techniques, and in the case of complex methodologies, have referred readers to the detailed technical explanations. In future studies, the analytical approach should be chosen based on the causal question that the study addresses.

Our causal model provides insight into previous research on the subject. Studies that have not included ozone in their models have estimated the combined effect of temperature on mortality through both pathways, whereas studies that have included ozone in their models have estimated the controlled direct effects of temperature on ozone. To our knowledge, no studies have yet estimated the natural direct or indirect effects, or controlled direct effects. Inference from all studies requires that causal assumptions of no unmeasured confounders, positivity, and consistency are met.

Future research could extend the approach employed here to questions that are beyond the scope of this commentary, including analysis of seasonal and regional patterns, inclusion of other air pollutants, and application to studies of morbidity. There is a need for epidemiologic studies to more clearly articulate their methods so that effect estimates from these studies can be applied correctly in risk analysis (Fann et al. 2011). Knowledge of whether an epidemiologic analysis investigated the total effect, the direct effect, or the indirect effect of temperature on mortality is particularly important for the estimation of future health risks of climate change.
